# On the Necessity of Consciousness for Sophisticated Human Action

**DOI:** 10.3389/fpsyg.2018.01925

**Published:** 2018-10-08

**Authors:** Roy F. Baumeister, Stephan Lau, Heather M. Maranges, Cory J. Clark

**Affiliations:** ^1^School of Psychology, Faculty of Health and Behavioural Sciences, University of Queensland, Brisbane, QLD, Australia; ^2^Department of Psychology, Florida State University, Tallahassee, FL, United States; ^3^Department of Psychology, Durham University, Durham, United Kingdom

**Keywords:** consciousness, conscious thoughts, epiphenomenalism, freedom, belief in free will

## Abstract

In this essay, we aim to counter and qualify the epiphenomenalist challenge proposed in this special issue on the grounds of empirical and theoretical arguments. The current body of scientific knowledge strongly indicates that conscious thought is a necessary condition for many human behaviors, and therefore, consciousness qualifies as a cause of those behaviors. We review illustrative experimental evidence for the causal power of conscious thought while also acknowledging its natural limitations. We argue that it is implausible that the metabolic costs inherent to conscious processes would have evolved in humans without any adaptive benefits. Moreover, we discuss the relevance of conscious thought to the issue of freedom. Many accounts hold conscious thought as necessary and conducive to naturalistic conceptions of personal freedom. Apart from these theories, we show that the conscious perception of freedom and the belief in free will provide sources of interesting findings, beneficial behavioral effects, and new avenues for research. We close by proposing our own challenge via outlining the gaps that have yet to be filled to establish hard evidence of an epiphenomenal model of consciousness. To be sure, we appreciate the epiphenomenalist challenge as it promotes critical thinking and inspires rigorous research. However, we see no merit in downplaying the causal significance of consciousness *a priori*. Instead, we believe it more worthwhile to focus on the complex interplay between conscious and other causal processes.

## Introduction

Everyday experience furnishes the strong impression that conscious thoughts such as decisions, plans, and intentions play an important role in causing behavior. Epiphenomenalism challenges that impression. An epiphenomenon is defined as a byproduct of other processes that does not itself cause anything. In philosophy, epiphenomenalism proposes that human conscious thought has no causal influence on the physical world, including human action. Huxley (1874) compared human conscious thought to a steam whistle on an engine: something that results from the engine’s processes and may reveal something about the activities inside the engine – but, crucially, that has zero effect on propelling or steering the train. In this paper, we argue against the notion of conscious thought being just the steam whistle of an engine, and for the idea that conscious thought is an important part of the inner, causal machinery instead.

We concede that it is not obvious how mental events such as conscious thoughts and feelings could influence the physical world. However, it is likely that these unknowns largely reflect limits in knowledge of neuroscientific representations of consciousness. That neuroscience has yet to fully understand the neurological underpinnings of consciousness, in our view, indicates nothing about whether consciousness is causal. Rather, it provides all the more reason to study how and why consciousness evolved and seems inseparable from many of the most sophisticated human behaviors.

There are compelling philosophical objections to epiphenomenalism (see Robinson, 2015). But those arguments have been made before, and we are not philosophers, so they are not our focus. Instead, in this brief essay, we summarize a number of psychological objections to the epiphenomenalist view of consciousness. Like the epiphenomenalists, we reject the naïve everyday impression that conscious thoughts are wholly able to dictate actions – but unlike them, we also reject the opposite extreme view that conscious thought is a feckless epiphenomenon. Instead, we affirm the conclusion made by a systematic review of experimental studies by Baumeister et al. (2011): Most human behaviors, especially most meaningful actions (thus excluding reflexes and the like), are the result of both conscious and unconscious processes, both of which are neurologically represented in the brain. By consciousness, we mean conscious thought, mental processes that take place within reflective consciousness and awareness and that are by default not functional without consciousness. Conscious thoughts are distinguished from other mental processes by the fact that the person can report on them to others, and indeed Baumeister and Masicampo (2010) proposed that the ability to communicate thoughts to other people was a main reason for consciousness to evolve. The human mind is adaptively designed to benefit from both conscious and unconscious processes, typically operating in concert. Consciousness is a necessary requisite to complex human behavior, even if its effects are mostly indirect. Hence, we shall conclude that the way forward is to understand how conscious and unconscious processes work together interactively to cause behavior.

## Consciousness Causing Behavior

The question of whether consciousness causes behavior was answered with a resounding no by Huxley’s (1874) “steam whistle” hypothesis. Many researchers have also interpreted Libet’s (e.g., Libet’s 1985, 2004) work as favoring an epiphenomenalism of the mind and conscious will (e.g., Wegner, 2002), though this conclusion is not actually supported by that work (e.g., Mele, 2009; Papanicolaou, 2017). Libet’s results might question the proximal power of conscious thought (i.e., direct action control) but still leave open distal effects of conscious thought, as demonstrated by Libet’s (1985) own model of a conscious veto-capacity. Seeming to support the epiphenomenalist position that consciousness lacks causal force, some psychological researchers such as (e.g., Bargh, 1997, 2006; Bargh and Chartrand, 1999) have proposed that all behavior is caused by unconscious processes and that the role of conscious thinking is, if not utterly zero, at least quite minimal and peripheral.

But does consciousness really play no causal role? It seems wildly implausible that human conscious thought evolved purely as a side effect, with no adaptive benefits depending on its ability to guide behavior. Conscious thought is observed most obviously among humans, the one species that also happens to be taking over the planet. Most likely, conscious thought is one reason for humankind’s success. Conscious thought facilitates complex human culture, our most adaptive survival strategy (Baumeister, 2005) – it promotes coherent communication (Baumeister and Masicampo, 2010), the spread of essential information through the group (Baumeister et al., 2018b), and consideration of the future and subsequent planning (Baumeister et al., 2018a).

Moreover, consciousness seems to depend on the interplay between cerebral cortex and other brain regions. The activity of these extensive brain regions is metabolically costly (Baars and Gage, 2010; Baars, 2012; Howarth et al., 2012). Conscious thought has considerable information processing costs due to its limited capacity, from which many disadvantages can result (e.g., distractibility, slow reactions; see Baars, 2012). It is implausible to assume that such a costly adaptation would have been selected for if it did not confer large compensatory benefits to the self and the group.

When understanding consciousness as a necessary part or condition of processes that lead to functional outcomes, consciousness qualifies as a cause in Mackie’s (1974) INUS model of causation. Real-world phenomena (such as social behavior) consist of complex constellations in which many factors contribute to one effect. A causal factor is therefore defined as an Insufficient but Necessary part of an Unnecessary but Sufficient condition (INUS). This means, for instance, that the conscious intention to murder someone alone is not sufficient for murder to happen. Many other conditions come into play as well to sufficiently create the effect (murder), such as a suitable weapon, a situational opportunity, and the lack of adequate self-protection by the victim. But the intention is often one crucial factor, without which the murder would not take place. Hence, following Mackie’s (1974) analysis, consciousness represents an epistemologically necessary – and thus causal – part of many complex behaviors.

## Varieties of Causation by Conscious Thoughts

Taking up the challenge to respond to the ephiphenomenalists, from Huxley (1874) up to the many who mistakenly think Libet proved conscious thought to lack causal power, Baumeister et al. (2011) surveyed the research literature in psychology for evidence of conscious causation of behavior. Scientists recognize experiments as the best method for testing causal theories. Therefore, Baumeister et al. (2011) searched for experiments that manipulated some conscious thought or feeling as independent variable and subsequently measured behavior as dependent variable. Any significant effects would indicate that the conscious thought or feeling caused behavior.

Their search uncovered a huge amount of evidence indicating conscious causation of behavior. Interested readers are referred to that review (Baumeister et al., 2011), and here, we merely summarize briefly the kinds of evidence for conscious events, thoughts, and feelings causing behavior.

Mentally (consciously) imagining specific actions increases the likelihood that they will be enacted. Mentally practicing sports and other skills improves subsequent performance. Some expectancies may be unconscious, but still, conscious expectancies do alter behavior. Deliberate planning changes and usually improves behavioral outcomes. Consciously forming specific “implementation intentions” (Gollwitzer, 1999) increases the likelihood that the desired action will be performed. How people think about recent events, and how much they think about them, alters how they act. Manipulating how people consciously think about themselves, for better or worse, has been shown to produce a wide range of consequences. People have many automatic responses, but conscious effort can block and override them. Changing the conscious mental framing of a task, including things like changing what one’s goal is, has been shown to alter multiple behaviors.

Several groups of research findings emphasized social uses of consciousness, and indeed there is a reasonable case that human consciousness evolved to serve interpersonal functions (Baumeister and Masicampo, 2010). Mentally simulating another person’s perspective does wonders for interpersonal harmony, though it can also be used strategically to gain competitive advantage. In negotiation (a form of interactive decision that is unique to humankind), consciously simulating the other side’s perspective has been shown to be widely influential and mostly helpful. Furthermore, empathy and perspective taking are important assets for ethical reasoning and moral behavior, especially in the current globalized social culture. It can be argued that these processes involve conscious thought and even need conscious awareness to come into being in the first place (see Haladjian and Montemayor, 2016).

Conscious thought is also necessary for logical reasoning (e.g., DeWall et al., 2008). Conscious beings use logical reasoning to their benefit. Scientific theories, for example, are mental constructs that depend on logic and have vastly influenced the physical world we live in Popper and Eccles (1977).

Another such phenomenon requiring conscious thought is talking, that is, coherent verbal communication (Baumeister and Masicampo, 2010). Consciousness is apparently required for integrating words into sentences, stories, and more. Via speech, members of groups share information, build a collectively shared stock of information, make group plans and execute them, devise cultural rituals, cope collectively with novel threats and opportunities, and evaluate (and thus learn from) each other. Indeed, the advantages of talking would seemingly be alone sufficient to explain the evolutionarily adaptive advantage of consciousness. If two hominid groups competed, and only one of them could talk, the talkers would have a substantial advantage in planning, coordinating, group decision, and other factors. Hence, consciousness appears to be a necessary condition of these processes, therefore qualifying as a cause in Mackie’s (1974) INUS model of causation.

## What Conscious Thought Can and Cannot Do

Several crucial themes and limitations of consciousness were noted by Baumeister et al. (2011). In general, the effects of conscious thinking were indirect. The most direct interventions produced negative effects, like choking under pressure, which occurs because the performer tries to supervise consciously the execution of an automatized skill (Baumeister, 1984). Unconscious, automatic causes may often be the proximal causes of action – but conscious thought is a powerful upstream cause, and it seems the farther upstream, the better.

Nothing showed that consciousness can produce behavior by itself. Obviously, if the conscious mind decides to walk toward a certain direction, control is handed off to the unconscious processes of walking. The proximal execution of behavior as physical movement is mainly, perhaps even exclusively, unconscious and automatic. If consciousness has any causal impact, it is far upstream from the actual firings of nerve cells to activate muscles to move. Hence the conclusion that almost all human behavior stems from a combination of conscious and unconscious processes.

Consciousness was especially found to be necessary in situations that had multiple possible outcomes, the “matrix of maybe” (Baumeister et al., 2018a). The relevance of consciousness to such situations may reflect that people use their conscious thoughts to imagine various possible futures and to calculate how their own actions might lead to these good and bad outcomes. Hence they alter their behavior based on how they have mentally simulated various future outcomes.

## Free Action and Conscious Thought

Debates on the causal power of consciousness often become entangled with debates about human freedom of action. But these are different questions. It is entirely possible that conscious thoughts could have powerful and extensive causal impact in the complete absence of free will and free action.

Still, there is some overlap. Insofar as decision freedom exists, it presumably relies on conscious causation. Hardly anyone has seriously developed a theory of unconscious freedom (cf. Heisenberg, 2009). Most conceptions of naturalistic free will and decision freedom include conscious processing, like self-reflection, as a necessary component (Lau and Hiemisch, 2017; also Fromm, 1964; Johnson-Laird, 1988; Hájiček, 2009). A reflective process enables considering multiple possibilities, cognitive flexibility, and the chance to incorporate higher values and abstract meanings into guiding action. The epiphenomenalist challenge thus implies that freedom could be ruled out as well as conscious causation, if conscious thought should turn out to be causally trivial.

Epiphenomenalism might discourage researchers from investigating adaptive forms of human freedom in relation to laypersons’ conscious experiences of it. For example, our work has revealed a mismatch between philosophical theories about decision freedom and laypersons’ self-reports of decision freedom. Lau et al. (2015) reported a series of experiments measuring how free people felt while making various decisions. The experiments manipulated a host of variables that theorists have linked to free action: number and diversity of options, uncertainty about future outcome, competing reasons, (absence of) time pressure, lack of a clear best option, difficulty of deciding, and the like. These generally made no difference or in some cases detracted from feelings of freedom rather than increasing it. Instead, people reported feeling freest when they obtained a positive outcome with minimal effort.

In our view, these results raise interesting questions for free will research, especially on the alignment of theories of free choice and folk psychology. Presumably the free and conscious contemplation of options represents an evolutionarily new and powerful way of making good choices, one that especially facilitates coping with the ambiguities of complex cultural life. The conscious self can carry out decision processes that are beyond what simple, automatic processes can do. Free choice should therefore be slow, effortful, and difficult (Lau and Hiemisch, 2017). Yet, people experience it directly the opposite way – they seem to feel freest when they can get what they want, quickly and easily. This suggests that laypeople possibly misrepresent (and misunderstand) the nature of free choice. Although attaining desirable outcomes is a part of free actions (Stillman et al., 2011), people seem to fantasize that one should always get the best option and move easily from joy to joy. If that were possible, humankind would not seemingly need the sophisticated mental and brain apparatus for complex processing. To freely control and steer one’s life includes facing uncertainty and resolving incompatible demands. Ruling out conscious causation *a priori* (and thus ruling out human freedom) discourages this kind of work that raises important questions for understanding the human experience of freedom.

## Beliefs About Free Will

People’s beliefs about free will represent one conscious cognition, indeed one that can be manipulated by conscious thought processes (Vohs and Schooler, 2008; Alquist et al., 2013a,b; Shariff et al., 2014) and conscious contemplation of conscious thought processes (e.g., Clark et al., 2017; Vonasch et al., 2017). For example, belief in free will dwindles when people read arguments that it does not exist, that neural evidence of a decision precedes conscious awareness of that decision, or that conscious free choice is an illusion. Moreover, these beliefs have behavioral consequences (particularly, a variety of prosocial and antisocial consequences), thus providing further support for the causal efficacy of conscious thoughts (for reviews, see Baumeister and Brewer, 2012; Baumeister and Monroe, 2014). These findings are particularly relevant to epiphenomenalism, because they show that conscious beliefs in free will (something that epiphenomenalists would argue does not exist in the physical world) have behavioral consequences.

Why do people believe in free will? Experiments by Clark et al. (2014, 2017) confirmed one reason. They showed that people increase their broad beliefs in human free will when they wish to punish others and to justify their punitive responses. These fit Nietzsche’s (1889/1954) speculation that the notion of free will was invented to create human moral responsibility, so that people could justifiably blame and punish their neighbors for misdeeds. Societies made up of citizens who believed in free will and who were correspondingly harshly punitive presumably flourished in comparison to and in competition with societies that failed to punish misbehavior.

Thus, conscious beliefs in free will generally make people behave prosocially, and these beliefs are used to justify punishing people who behave antisocially. The implication is that both the belief in free will and the reality (such as it may be) of free will improve human interaction in ways that lead to broadly beneficial, adaptive outcomes.

## A Challenge to Epiphenomenalists

The special issue’s theme is the epiphenomenalist challenge to theories of conscious causation and free will. It claims that conscious thought has no causal power. We end by issuing a reciprocal challenge to the one set forth by this special issue. We challenge proponents of this view to conduct their experiments without relying on conscious causation (e.g., for instructions, informed consent). We think this is unfeasible because conscious processes are a necessary condition for many behaviors, particularly social ones. On a pragmatic note, it is likely not conducive to scientific discovery to rule out conscious causation *a priori*. How can one empirically study an alternate (and likely impossible) reality in which consciousness does not co-occur with its neural correlates?

If we proceed to explain behavior only in terms of neuronal events, like eliminative materialists call for, what exactly are the causal units or factors and on which level of resolution can we describe them (e.g., neuronal, atomic, subatomic)? A vast body of knowledge about the brain shows that “neural activity” can be understood as the activity of lobe regions, of clusters and networks, or as the workings and interplay of (billions of) single neurons. All of these levels of explanation can be causal, but the lower level correlates of consciousness are confounded with consciousness itself, which is also causal insofar as it is a necessary component of complex behaviors (see section “Consciousness Causing Behavior”). Conscious experience is a concise concept with pragmatic benefits for explaining human behavior. Imagine the absurdity of trying to furnish an explanation of the worldwide economic crisis of 2008–2009, or the First World War, or the rise of Islam, purely in terms of neuronal activity. As this reasoning should convey, we believe that the qualitative differences between mind and brain alone serve to counter and qualify the notion of an epiphenomenalism of mental events.

To be sure, we welcome the epiphenomenalistic challenge of this special issue as it inspires critical discussion and exciting new experiments. However, we believe that psychological phenomena are complex to such a degree that we should keep investigating all facets (conscious thought, unconscious cognition, brain mechanics). Instead of reducing one to the other *a priori*, we should focus on their interplay in the causation of experience and behavior.

## Conclusion

Everyday experience supports the impression that conscious thoughts guide and control behavior. By definition, one is not conscious of unconscious processes, and so one cannot easily appreciate them. Nevertheless, scientific research has confirmed the ubiquitous operation and importance of unconscious processes for human action. The exact causal sequences for conscious processing are similarly, mysterious, but nonetheless, as we have covered with ample research, including rigorous laboratory experiments, conscious thoughts also help cause behavior. Unlike the unrealistic and contrived scenario in Libet’s experiments, in which all uses of conscious thought were carefully ruled out (e.g., by instructions not to plan), we think ordinary human action relies heavily on conscious reasoning, conscious understanding, conscious explanation and communication, and the conscious processes of reaching agreement, operating in concert with various other levels of causation.

The human capacity for conscious thought, including conscious communication, is a major reason for the unprecedented sociocultural achievements of humankind. Rather than ruling out conscious causality *a priori*, future research may profit from adopting a more moderate position and aiming to elucidate how conscious and other causal processes complement each other in guiding human action.

## Author Contributions

RB, SL, HM, and CC conceived the paper. RB wrote the draft. SL, HM, and CC provided the additional content, ideas, literature, comments, as well as thorough revisions.

## Conflict of Interest Statement

The authors declare that the research was conducted in the absence of any commercial or financial relationships that could be construed as a potential conflict of interest.
